# Investigating fine and gross motor deficits in pediatric patients off therapy for acute lymphoblastic leukemia

**DOI:** 10.1080/07853890.2025.2477292

**Published:** 2025-03-13

**Authors:** Filip Jevic, Ross Andel, Monika Hrdouskova, Alena Kobesova

**Affiliations:** ^a^ Department of Rehabilitation and Sports Medicine, Second Faculty of Medicine, Charles University and University Hospital Motol, Prague, Czech Republic; ^b^ Third Faculty of Medicine, Charles University, Prague, Czech Republic; ^c^ Edson College of Nursing and Health Innovation, Arizona State University, Phoenix, Arizona, USA; ^d^ International Clinical Research Center, Brno, Czech Republic; ^e^ Institute for the Care of Mother and Child, Prague, Czech Republic

**Keywords:** Paediatric acute lymphoblastic leukaemia (ALL), motor skills assessment, Bruininks-Oseretsky test of motor proficiency (BOT-2 CF), motor performance deficits, rehabilitation in paediatric oncology

## Abstract

**Purpose:**

To assess motor performance among Czech paediatric off therapy patients of acute lymphoblastic leukaemia (ALL) and to compare their data with normative data.

**Methods:**

Thirty-nine off therapy patients (21 girls, 18 boys; aged 4–21 years) were evaluated using the Complete Form of the Bruininks-Oseretsky Test Second Edition (BOT-2 CF) approximately 1.5 years post-therapy cessation. Gross and fine motor skills were assessed. Normative data from BOT-2 CF served as the basis for comparison.

**Results:**

The total motor composite (*p* = .381, Cohen’s *d* = 0.14) and overall fine (*p* = .743; Cohen’s *d* = 0.05) and gross (*p*=.312; Cohen’s *d* = 0.16) motor performance were similar to the normative data. Motor deficits in manual coordination (*p* = .018; Cohen’s *d* = 0.45), strength and agility (*p* = .012; Cohen’s *d* = 0.51), manual dexterity (*p* < .001; Cohen’s *d* = 0.59) and running speed and agility (*p* < .001; Cohen’s *d* = 0.97) were identified, along with performance better than the established norms on fine motor integration (*p* = .048; Cohen’s *d* = 0.33) and bilateral coordination (*p* = .018; Cohen’s *d* = 0.47).

**Conclusion:**

The findings suggest nuanced motor skill outcomes in ALL off therapy patients, with both deficits and strengths observed. Comprehensive assessments are vital for tailoring rehabilitation strategies to address the varied impacts of ALL and its treatment on motor skills.

## Introduction

Acute lymphoblastic leukaemia (ALL) is the most common childhood malignancy, with survival rates exceeding 90% [1]. The main treatment lasting from 2 to 3 years depending on the patient’s stratification risk is based on application of corticosteroids and chemotherapy [2]. With increasing survival rate, the early detection and treatment of the therapy side effects become more important [3]. In all, 35–40% of ALL survivors report late side effects affecting functional, cognitive, and/or physical domains [4]. Late side effects of corticosteroid therapy and chemotherapy may involve myopathy [5], osteonecrosis [6], osteoporosis [7], Vincristine induced polyneuropathy [8], corticosteroid obesity [9] and cardiomyopathy [10].

Motor impairments can include proximal and distal muscle weakness [3,11–16], reduced ankle range of motion (ROM) [12,14,17], balance problems [12,17–21], gait disturbances [3,15,17,21] and decreased aerobic endurance [11,13], all of which may impact motor proficiency within both gross [22,23] and fine [24–28] motor skills as well as overall motor proficiency [11,29–32]. For assessing motor proficiency, different tools have been applied. Using UQAC-UQAM (Université du Québec à Chicoutimi (UQAC) and Université du Québec à Montréal (UQAM)) testing battery, Leone et al. [23] report that nearly 50% of ALL survivors scoring below 15th percentile in gross motor skills such as speed, agility, balance, coordination and reaction time. Among survivors, 53% experienced a decline in body coordination to below average levels and 27% in strength and agility using Bruininks-Oseretsky Test Second Edition (BOT-2) [22]. Finally, 54% of children from the monitored group scored below 15th percentile in Ball Skills assessed by Movement Assessment Battery for Children – Second Edition (MABC2) [11]. In addition, handwriting, a fine motor skills component, was found to be impaired in 25% of ALL survivors at 2+ years post-treatment [24].

Studies using MABC2 to assess manual dexterity have yielded conflicting results. Reinders-Messelink [24] reported significantly worse manual dexterity in ALL survivors but van Brussel et al. [11] reported that ALL participants scored above the 15th percentile, that is within the norm, using the same test. Assessing overall motor proficiency with MABC2 and BOT-2 Short Form (BOT-2 SF) de Luca [29] found mean total scores of all 37 ALL survivors 0–12 months post-treatment to fit within the average, while Hartman [30] found only 58% of their sample to be within the average using MABC2, with the remaining participants significantly below or well below average. Ramchandren [31] reported that only 5.7% of survivors scored below the average with none scoring substantially below average on BOT-2 SF, while Tay [32] found 13% of survivors to fit bellow or substantially below average on this test. Variations in motor proficiency results among ALL survivors using MABC2 and BOT-2 SF in different studies can be due to factors like sample differences (age, presence of neuropathy or avascular necrosis, treatment protocol arm, radiotherapy inclusion); time since treatment (changes in motor skills due to recovery or new challenges); research design and methodology (study type, measurement protocols, statistical methods); heterogeneity in ALL treatment protocols (chemotherapy regimens, supportive care); and individual variability (pre-existing conditions, genetics, response to treatment).

Still, the BOT-2 is considered a reliable and valid assessment tool for evaluation of the individual’s motor skills [33]. The test contains two forms, complete form (CF) consisting of 53 items across all subtests and short form (SF) consisting of 14 items selected from the full BOT-2 test [34]. BOT-2 SF was used in several studies assessing ALL survivors [29,31,32]. Some researchers used some subtests of BOT-2 CF [20–22] (such as balance or strength subtest) in ALL patients assessment, but there is no study reporting the results of the complete BOT-2 CF in ALL child survivors. Building on previous research, the aim of our study was to assess motor performance of Czech ALL child off therapy patients using BOT-2 CF, comparing ALL child off therapy patients data with the normative data of healthy children reported in BOT-2 CF manual [33]. We expected that ALL patients would score lower compared to normative data overall, but also that individual domains of both gross and fine motor skills would differ measurably. In addition to knowledge gained from existing research, our personal clinical experience working with paediatric ALL off therapy patients dictates that spending a long time in bed may be offset not only by the use of electronic devices but also by engaging in various small manual tasks. This could hypothetically lead to better performance in certain fine motor domains compared to age- and gender-matched norms.

## Materials and methods

### Setting and participants

**Figure 1.**
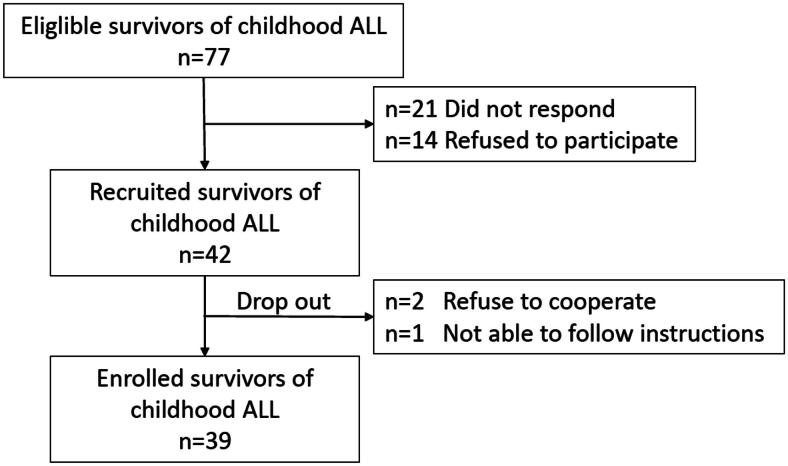
Flowchart of patient recruitment.

This cross-sectional, single-centre study of off therapy patients of childhood acute lymphoblastic leukaemia (ALL) was conducted from 22 November 2020 to 16 December 2021. Participants were recruited from the Department of Paediatric Haematology and Oncology, Second Faculty of Medicine, Charles University in Prague and Motol University Hospital. Eligible participants were identified from the oncology registry. Inclusion criteria were (1) ALL as primary malignant disease, first occurrence; (2) treatment followed the AIEOP-BFM ALL 2009 protocol for both standard-risk and high-risk arms; (3) completed ALL treatment more than 1 month and less than 4 years before the study enrolment; (4); age 4–21 years. Exclusion criteria included (1) relapse of ALL, as the treatment protocols for relapsed patients differ significantly from those in remission, potentially introducing additional variables that could affect motor outcomes; (2) the presence of a neurologic, genetic, or developmental disorder prior to the ALL diagnosis; (3) cranial radiotherapy during treatment, which may impact neurodevelopment and independently affect motor function [35]; and (4) Haematopoietic Stem Cell Transplantation (HSCT), due to potential complications following transplantation that could independently affect motor function, making it challenging to isolate the specific effects of leukaemia treatment. Out of 77 eligible patients, 42 participants (54.5%) were recruited, and 39 eventually participated in the study, as illustrated in Figure 1. Some patients did not respond to the study recruitment efforts due to a variety of reasons including inaccurate contact information, lack of interest in participating, or family circumstances that prevented their participation. Given that survivors are typically considered individuals who have been off treatment for at least 5 years [4,36], and the mean time since the cessation of therapy for our participants is 1.5 years, we refer to them as ‘off therapy patients’ in this study. All participants received vincristine, dexamethasone, and intrathecal methotrexate according to the specified treatment protocol (standard risk or high risk). Minor variations in cumulative vincristine doses occurred due to neurotoxicity in three patients: one standard-risk patient received a reduced cumulative dose of 6 mg/m^2^ and was switched to vinblastine, while two high-risk patients received a cumulative dose of 15 mg/m^2^ instead of the protocol standard of 18 mg/m^2^. Dexamethasone doses were consistent with protocol guidelines for all participants.

The study was conducted in accordance with the Declaration of Helsinki, approved by the Ethics Committee Ethics Committee of Third Faculty of Medicine, Charles University, Prague, Czech Republic. Written informed consent was obtained from the parents or legal guardians of the participants.

### Procedures and measurements

Participants were assessed during their standard ALL follow-up visit to the oncology clinic. The following clinical data were obtained from the medical records (see Table 1): Age at the time of diagnosis, time since the end of maintenance therapy, treatment protocol, cumulative doses of Vincristine, prednisone, dexamethasone and intrathecal methotrexate, and the presence of avascular necrosis. Height (cm) and weight (kg) were recorded for all participants before starting the evaluation procedures. For evaluating motor proficiency the Bruininks-Oseretsky Test of Motor Proficiency-second edition (BOT-2 CF) was performed following standard procedures as suggested by the test manual [33]. The BOT-2 is a widely used, reliable tool for evaluating motor proficiency in children and adolescents aged 4 to 21, assessing several domains such as fine motor control, manual coordination, body coordination, and strength and agility. It is available in two forms: the complete form (CF) with 53 items and the short form (SF) with 14 items. We chose to use the CF because it provides a more comprehensive and detailed assessment of motor skills, allowing for greater precision in evaluating deficits. This was particularly important in our study, as we aimed to capture the full range of motor skills in our patient sample. Multiple studies have demonstrated its strong psychometric properties. The BOT-2 has shown excellent internal consistency, with Cronbach’s alpha coefficients ranging from 0.89 to 0.92 [37,38]. Test-retest reliability has been reported as excellent, with intraclass correlation coefficients (ICC) of 0.99 for both the complete form and specific subtests [37,38]. Inter-rater reliability has also been found to be high (ICC = 0.88 − 0.99)[38,39]. The BOT-2 has demonstrated good validity, including concurrent validity with other motor assessments [40].

**Table 1.** Sample characteristics; *n* = 39.

	Mean or n	SD	Range
Age at diagnosis	6.7	4.1	1.3 − 16.6
Age at assessment	10.1	4.1	4.5 − 20.8
Girls/boys	21/18	--	--
Weight, in kg	37.2	16.0	15.2 − 75.3
Height, in m	1.4	0.2	1.0 − 1.8
BMI	18.4	3.8	13.4 − 28.3
Years since end of maintenance therapy	1.5	1.2	0.0 − 4.0
Cumulative dose of MTX, in mg	158	51.3	88.0 − 264.0
Cumulative dose of Prednisone, in mg/m^2^	1732.7	217.4	410 − 1767.0
Cumulative dose of Dexa, in mg	398.9	255.6	236.5 − 828.8
Ped-mTNS score	3.4	4.5	0 − 21.0
High risk, n yes	11	--	--
Neuropathy, n yes	7	--	--
Avascular necrosis, n yes	5	--	--

BMI: body mass index; Dexa: dexamethasone; MTX: methotrexate; Ped-mTNS: Pediatric modified total neuropathy score; SD: standard deviation.

Paediatric modified total neuropathy score (Ped-mTNS) is a valid and reliable tool to assess chemotherapy-induced peripheral neuropathy in children with non-CNS cancers [41], it captures sensory, motor and autonomic symptoms, light touch, pin and vibration perception, muscle strength of distal musculature and deep tendon reflexes and was assessed in the same way in each child following standard assessment protocol. Studies have demonstrated its construct validity, feasibility, and ability to differentiate between patients and healthy controls [42–44]. The Ped-mTNS has shown good internal consistency and can be completed in under 10 min [42,43]. The Ped-mTNS is recommended for assessing Vincristine-induced peripheral neuropathy in children, with a score of 5 indicating neuropathy. The threshold of 5 points was also used in our study to determine neuropathy [45].

Both of the tests were performed by the same trained physical therapist (PT). Each child was assessed individually in a PT’s office under the same assessment conditions (daytime, room temperature, room equipment). The procedure took approximately 60–70 min.

Osteonecrosis was identified exclusively in the lower extremities of the participants, specifically in the hip, knee, distal femur, talus, and calcaneus. These findings were based on X-ray examinations, which were performed at the site of reported pain, however, the staging of osteonecrosis was not conducted. Our study focused on the presence of osteonecrosis rather than its specific stage classification.

### Statistical analysis

We present characteristics of our sample *via* descriptive information. Before analyses, collected data regarding motor efficiency were examined for normality of score distributions. All variables were in the acceptable range for skewness and kurtosis (values consistently below ±0.80) except for manual dexterity which showed positive skewness (skewness 1.54). This was rectified by removing one observation/outlier that was about four standard deviations above the mean (resulting skewness <0.80, commensurate with other measures).

Initially variables were descriptively described using correlation between each independent variable and one of the three dependent variables presented as Pearson Product Moment coefficients when both variables were continuous and Point-Biserial coefficients when one variable was continuous and the other was binary.

All composites and individual measures of motor proficiency were examined using a one-sample t-test, whereby the z-score-standardized means and standard deviations from our sample were compared to international normative data [33]. SAS (SAS Institute, Cary, NC) procedure T-TEST was used with the *p*-value set at a two-tailed .05. In all, we calculated 12 t-tests across the 12 individual motor proficiency measures, presumably increasing our chances of committing a Type I error. Therefore, *p*-values for individual tests were subsequently adjusted for multiple comparisons using the Holm-Bonferroni method [46], and these *p*-values are reported. Cohen’s d [47] was calculated by subtracting the standardized sample mean from the normative mean over sample standard deviation.

## Results

Sample characteristics are shown in Table 1. The 39 participants were on average 10.1 ± 4.1 years old at assessment with an about even distribution by sex. In comparison, the 38 patients who were eligible but did not participate were similar to those who participated in terms of age at contact (i.e. assessment) (9.5 ± 4.3 years old; t[75] = 0.56, *p* = .578) and sex (19 males/19 females; χ^2^[1] = 0.11, *p* = .736). To estimate differences in severity, we used assignment into a high vs. low risk protocol as other measures were not available for those who did not participate in our study. In all, 11 (28%) participants and 5 (13%) non-participants were in the high-risk protocol, with the difference in proportions being non-significant, χ^2^[1] = 2.64, *p* = .104.

The BMI of the participants was within the normal range within the context of the age and gender distribution of the participants. The participants were about 1.5 ± 1.2 years removed from ending maintenance therapy. Of the 39, 11 were in high-risk arm of treatment, 7 expressed with neuropathy and 5 with avascular necrosis. On average, participants received approximately 10 physical therapy sessions during the follow-up period, with variations in frequency and duration based on individual needs and clinical recommendations.

Table 2 shows intercorrelations between the three motor composites and participant characteristics. Total motor composite correlated negatively with Ped-mTNS scores and avascular necrosis, and gross motor composite correlated negatively with age at assessment, age at diagnosis, Ped-mTNS scores, neuropathy, and avascular necrosis.

**Table 2.** Intercorrelations between motor skills composites and participant characteristics.

	Total MC	Fine MC	Gross MC
Age at diagnosis	−.29	−.13	−.40*
Age at assessment	−.23	−.09	−.34*
Girls/boys	−.26	−.27	−.23
Weight, in kg	−.13	−.02	−.22
Height, in m	−.15	.00	−.28
BMI	.02	.05	−.03
Years since end of maintenance therapy	.18	.08	.18
Cumulative dose of MTX, in mg	−.01	.15	−.19
Cumulative dose of Prednisone, in mg/m^2^	−.09	−.06	−.11
Cumulative dose of Dexa, in mg	.20	.30	.05
Ped-mTNS score	−.32*	−.21	−.39*
High risk, n yes	.16	.27	.00
Neuropathy, n yes	−.31	−.21	−.37*
Avascular necrosis, n yes	−.34*	−.20	−.43*

Notes. Pearson correlation coefficients are shown. *p<.05.

BMI: body mass index; Dexa: Dexamethasone; MC: motor composite; MTX: methotrexate; Ped-mTNS: pediatric modified total neuropathy score.

The main results are shown in Table 3. None of the scores on the composite measures deviated significantly from the established norms. With respect to individual tests, the participants performed worse relative to the norms on manual coordination (t(38) = -2.84, *p* = .018; Cohen’s *d* = 0.45), strength and agility (t(38) = −3.18, *p* = .012; Cohen’s *d* = 0.51), manual dexterity (t(37) = −3.61, *p* < .001; Cohen’s *d* = 0.59), and running speed and agility (t(38) = −6.03, *p* < .001; Cohen’s *d* = 0.97). The participants performed better than the norms on fine motor integration (t(38) = 2.04, *p* = .048; Cohen’s *d* = 0.33) and bilateral coordination (t(38) = 2.94, *p* = .018; Cohen’s *d* = 0.47).

**Table 3.** Comparison of participant performance to established norms.

	Mean	SD	Norm	t-test	DF	*p*-value	Cohen’s d
Composites							
Total Motor Composite	48.62	9.75	50.00	−0.89	38	.381	0.14
Fine Motor Composite	49.49	9.70	50.00	−0.33	38	.743	0.05
Gross Motor Composite	48.28	10.48	50.00	−1.02	38	.312	0.16
Fine Motor Control	52.92	9.52	50.00	1.92	38	.063	0.31
Manual Coordination	46.05	8.70	50.00	−2.84	38	.018	0.45
Body Coordination	53.03	10.24	50.00	1.85	38	.073	0.30
Strength and Agility	44.92	9.98	50.00	−3.18	38	.012	0.51
Subtests							
Fine Motor Precision	15.85	4.39	15.00	1.20	38	.236	0.19
Fine Motor Integration	16.72	5.26	15.00	2.04	38	.048	0.33
Manual Dexterity	12.76	3.82	15.00	−3.61	37	<.001	0.59
Upper-Limb Coordination	13.85	4.47	15.00	−1.61	38	.115	0.26
Bilateral Coordination	17.05	4.35	15.00	2.94	38	.018	0.47
Balance	15.05	4.99	15.00	0.06	38	.949	0.01
Running Speed and Agility	10.49	4.67	15.00	−6.03	38	<.001	0.97
Strength	14.97	4.64	15.00	−0.03	38	.973	0.01

Notes. *p*-values for individual tests are corrected for multiple comparisons using the Holm-Bonferroni method.

SD: standard deviation; DF: degrees of freedom.

Finally, we present the number of participants who performed at least 1 standard deviation (SD) below the normative average on our motor performance measures, overall and by neuropathy and avascular necrosis status (see Table 4). In general, participants performed well on the Fine Motor Control composite (within 1 SD), which includes the Fine Motor Precision and Fine Motor Integration subtests. However, they performed relatively poorly on the Manual Coordination composite, particularly on the Manual Dexterity subtest, and the Strength and Agility composite, particularly on the Running Speed and Agility subtest (see Table 4).

**Table 4.** N of participants *at/above* vs. *Below* 1SD below normative average overall (*n* = 39) and with neuropathy (*n* = 7) or avascular necrosis (*n* = 5).

	All		With neuropathy		With necrosis	
	At/above	Below	At/above	Below	At/above	Below
*Composites*						
Total Motor Composite	32	7	3	4	2	3
Fine Motor Composite	36	3	6	1	5	0
Gross Motor Composite	33	6	4	3	2	3
Fine Motor Control	37	2	7	0	5	0
Manual Coordination	30	9	3	4	3	2
Body Coordination	34	5	4	3	3	2
Strength and Agility	28	11	3	4	1	4
*Subtests*						
Fine Motor Precision	36	3	7	0	5	0
Fine Motor Integration	35	4	7	0	4	1
Manual Dexterity	30	9	1	6	1	4
Upper-Limb Coordination	33	6	5	2	4	1
Bilateral Coordination	35	4	4	3	3	2
Balance	34	5	4	3	3	2
Running Speed and Agility	21	18	2	5	1	4
Strength	36	3	5	2	3	2

## Discussion

The aim of our study was to present a detailed assessment of motor performance of young Czech ALL off therapy patients. The total motor composite and overall fine and gross motor performance were similar to the normative data. However, the patients performed significantly worse in specific (detailed) parts of both fine and gross motor performance such as manual dexterity, manual coordination, running speed and agility, and strength and agility. On the contrary, they scored significantly better in fine motor integration and bilateral coordination compared to the normative data. To the best of our knowledge, this is the only study reporting the complete form of BOT-2 test to detailed assessment of both fine and gross motor performance in a paediatric population of ALL off therapy patients.

Our results for overall motor performance (average TMComp standard score = 48.6 ± 9.8) are similar to the previously published studies using BOT-2 SF, including a study with 101 Malaysian children survivors all of whom were 2+ years after therapy (49.2 ± 8.8) [32] or a study with 37 Australian children survivors 0–60 months off treatment (51.0 ± 8.2) [29]. In other words, none of these studies found significant differences between the ALL survivors and the norms. However, the comparison between the studies applying short and long form of BOT-2 has some constraints. Although high reliability and strong correlation between BOT-2 CF and BOT-2 SF have been reported [33,37], it is important to acknowledge that discrepancies in estimating proficiency levels exist [48–51]. In our study, most participants performed within the normal range on BOT-2 (29 out of 39 = 74.4%), which matches the previous published data by Ramchandren [31] (75.7%) and by Tay [32] (73.3%). Therefore, there is substantial evidence that young ALL survivors are not worse in overall motor performance than healthy children.

### Gross motor deficits

Performance on the Gross Motor Composite in our study corresponds to normative data and most (71.8%) of our participants fit within the average in overall gross motor performance. Still, we found significant deficits in the Strength and Agility composite (*p* = .012, Cohen’s *d* = 0.51) and in the Running Speed and Agility subtest (*p* ˂ .001, Cohen’s *d* = 0.97). On the other hand, the Strength subtest (the second part of the Strength and Agility motor area composite) was right at the norm (*p* = .973, Cohen’s *d* = 0.01). This means that the main deficit of our ALL off therapy patients is the Running Speed and Agility Performance, and it is substantial enough where it appears to affect the results for the Strength and Agility composite even though the participants’ strength otherwise seems to be intact. Agility, i.e. the ability to rapidly change the position of the body in space with speed and accuracy [52], is necessary for performing complex multidirectional physical tasks [53]. Agility is a complex skill combining strength with, stability, reactivity, joint flexibility, reflexes and fluidity of movement. Of the 39 participants in our study, 7 had Vincristine-induced peripheral neuropathy, which could disturb the agility skills [54]. Also, five participants (13%) suffered from lower limb osteonecrosis located at the hip, knee, distal femur, talus, and calcaneus. This severe and painful late side effect of ALL treatment, which reduces mobility [6,55,56], also impacted the running and agility subtests. Deficits in running speed, agility, and strength have been reported in ALL survivors previously by Leone et al. [23], and Wright et al. [12].

Another important and frequently discussed part of the Gross motor skills is balance. Varedi et al. [19] reviewed published studies on balance during or after treatment for ALL, and concluded that survivors may experience short or/and long-term balance difficulties. However, in our study, the balance subtest scale score was within the normal and only 5 out of 39 participants (12.8%) were below average. One possible reason for the relatively low occurrence of balance disturbances in our study could be the exclusion of subjects who underwent cranial radiation therapy (CRT), which several authors have identified as a cause of balance impairment [13,19,57].

### Fine motor deficits

Although the mean Fine Motor Composite (49.5 ± 9.7) of our participants is within the norm and 71.5% of our participants fit within the average in overall fine motor performance, we found significant deficits in manual coordination composite (*p* = .018, Cohen’s *d* = 0.45) and in manual dexterity subtest (*p* ˂ .001, Cohen’s *d* = 0.59). On the other hand, in fine motor integration subtest and bilateral coordination subtest, our participants performed better than the normative data (*p* = .048, Cohen’s *d* = 0.33, and *p* = .018, Cohen’s *d* = 0.47, respectively). The contrast between overall fine motor skills and specific deficits suggests that average performance can mask specific areas of weakness. Fine motor integration involves the coordination of small muscle movements in the hands and fingers in unison with the movement of the eyes such as writing, drawing, using scissors, building with small blocks, using utensils or typing on a keyboard. Bilateral coordination refers to the ability to use both sides of the body simultaneously in a coordinated way such as catching and throwing a ball, clapping hands, or bouncing a ball with both hands. These activities are often used within occupational therapy and hospital educational settings to train fine motor integration skills. This maybe the reason why our group scored well in these subtests.

These results may indicate the need for a more nuanced approach to evaluating fine motor skills. While some motor skills may be impacted, others can remain intact or even improve which can lead to more focused and effective therapies and educational strategies. Hanna et al. [58] evaluated fine motor control (FMC) in children with ALL during maintenance therapy and concluded that 67% of these children experience fine motor difficulties. Also, according to Tejeda-Castellanos et al. [59], fine motor impairments are common in children with ALL during the maintenance phase, and early identification of these impairments is crucial for prompt rehabilitation. Unlike previous studies [58,59] that evaluated children during maintenance therapy and reported fine motor impairments, our study presents data collected 1.5 years after the cessation of therapy. This suggests that the quality of motor function may vary post-therapy: while some children’s motor functions are within normal ranges, others exhibit persistent deficits (manual coordination and dexterity), and intriguingly, some even surpass the norm (fine motor integration, bilateral coordination).

When comparing the results of various studies using BOT-2 we must consider the aspect of control group. While some studies compare the patient group to an age- and sex-matched control group [13,14,21,25,57], others use age- and sex-matched norm values for comparison [11,23,26,29,31,32]. This methodological approach can significantly influence outcomes. Age- and sex-matched controls may not exhibit typical motor skills. For example, Hanna et al. [58] used controls who demonstrated superior performance compared to the normative population, with only 3% falling below average. This discrepancy raises questions about the comparative significance of their FMComp scores against those of ALL patients, suggesting the control group may not accurately represent the general population’s fine motor skills. Unlike Hanna et al. [58], we compared the results of our 39 ALL subjects directly with BOT-2 standard norms. Our participants’ age range is broad, spanning from 4.5 to 20.8 years. However, the fact that BOT-2 provides normative data for sex-matched individuals aged 4 to 21 years increases the validity of our results.

Handwriting is a key aspect of fine motor performance. While we found no significant difference between our ALL off therapy patients and the norms in scores on the Fine Motor Precision subtest, which assesses handwriting and drawing, Reinders-Messelink et al. [24] reported that 25% of ALL survivors experience handwriting difficulties more than 2 years post-treatment, with younger children requiring more time and demonstrating less fluency than older ones. Goebel et al. [25] found impairments in speed, automation, and variability in drawing and handwriting in ALL off therapy patients 3.5 years after treatment. While the BOT-2 evaluates handwriting and drawing within its Fine Motor Precision and Integration subtests, it does not assess speed.

In light of our findings, implementing preventive programs focused on motor skill development, particularly for children identified as being at higher risk for motor deficits, is essential. Health professionals should consider designing targeted interventions that address areas with pronounced deficits, such as fine motor skills, manual dexterity, and agility. Early, structured activities and therapies can support motor development, reduce the long-term impact of treatment-related deficits, and potentially enhance quality of life for ALL survivors. Future studies could also investigate the efficacy of these preventive strategies to guide best practices in rehabilitation for this population.

This study has some limitations. We were able to enrol only about 50% of all eligible ALL off therapy patients which could lead to bias. In addition, we were unable to assess the severity of ALL in the eligible patients who did not participate other than through a comparison between high/low risk protocol assignment. The age range of participants was relatively broad, although this should not have influenced the individual results (the normative data of BOT-2 CF are also broad and age dependent). Still, our results could obscure potential specific age-dependent motor deficits.

While patients with relapses or those undergoing HSCT were excluded from this study due to the complexities of their treatment protocols and potential confounding effects on motor outcomes, we acknowledge that including these patients in future research may provide valuable insights into the full spectrum of motor sequelae associated with ALL treatment. Therefore, we recommend that future studies consider including these groups to further explore their unique motor deficits and long-term outcomes. Additionally, incomplete data on certain patient variables, such as specific therapies, duration of hospitalizations, and significant acute episodes like sepsis, were not analyzed. Future studies should aim for comprehensive inclusion of these variables to better elucidate their potential impact on motor outcomes in paediatric ALL survivors. Lastly, our findings should be interpreted with consideration of the rehabilitation services provided to participants. While the aim of this article was not to assess the impact of rehabilitation on motor outcomes, it is possible that these services influenced the results. On average, participants received approximately 10 physical therapy sessions, though the intensity, frequency, and total number of sessions varied based on indi­vidual needs, symptoms, and treatment phases. Additionally, all participants were advised to engage in moderate to light intensity physical activity for 30*–*50 min, three times per week. Future research should focus on systematically evaluating the effects of rehabilitation interventions on motor performance in paediatric ALL patients. Moreover, an analysis of sport re-entry among ALL survivors, including factors such as frequency of training, types of sport activities, and performance in Physical Education, could provide valuable insights into their functional recovery.

## Conclusion

Czech ALL off therapy patients exhibit motor deficits in specific areas of both gross and fine motor performance. Running speed and agility were the most affected parts of gross motor performance, while manual dexterity deficits were noted in fine motor performance. In contrast, fine motor integration and bilateral coordination were superior compared to normative data. To support motor recovery, health professionals should focus on early interventions that target these specific deficits during therapy, particularly in patients at higher risk, such as those with Vincristine-induced neuropathy or lower limb osteonecrosis. A structured, gradual sport re-entry program, focusing on improving strength, agility, and coordination, may be beneficial. Future studies should continue to explore motor deficits using comprehensive assessments like BOT-2 CF, which provides more detailed norms and may yield more accurate insights compared to BOT-2 SF.

## Data Availability

The datasets used and/or analyzed during the current study are available from the corresponding author upon reasonable request.
